# Supplementary figures and images for: Bacterial-archaeal co-occurrence in honey bee gut microbiomes across host species and management regimes
Source: PLoS One. 2026 Jan 21;21(1):e0339926. doi: 10.1371/journal.pone.0339926 (PMC12822932; doi:10.1371/journal.pone.0339926)

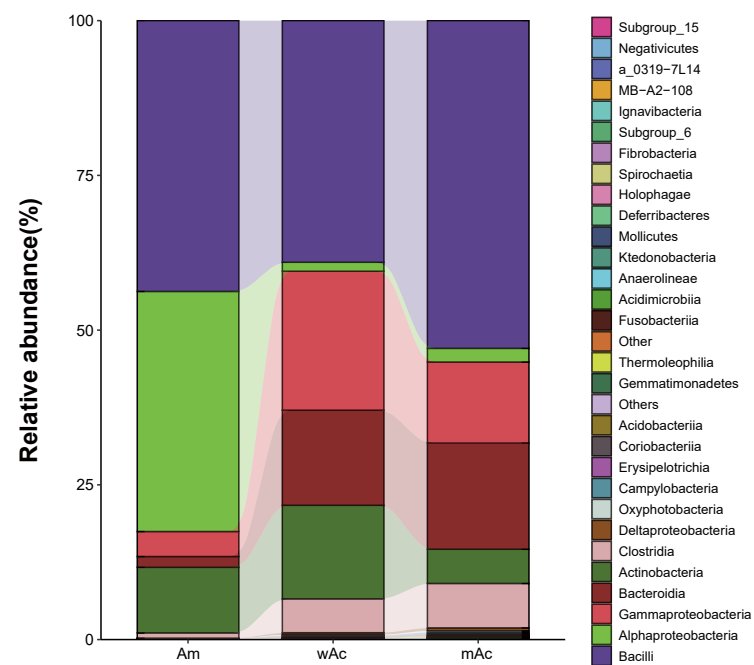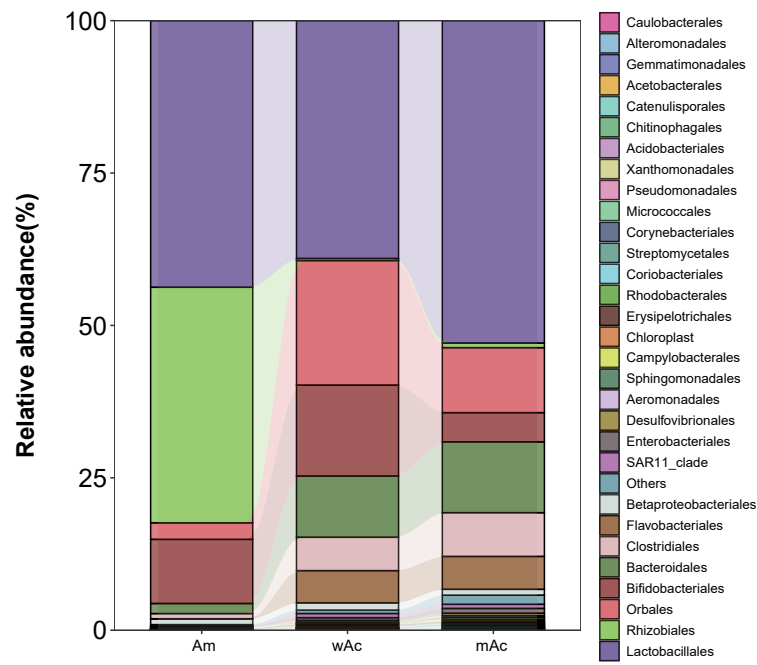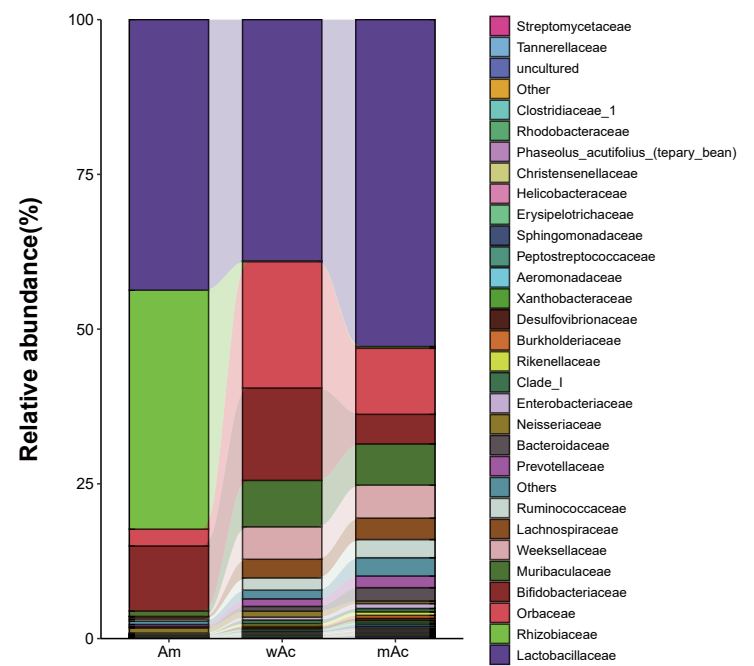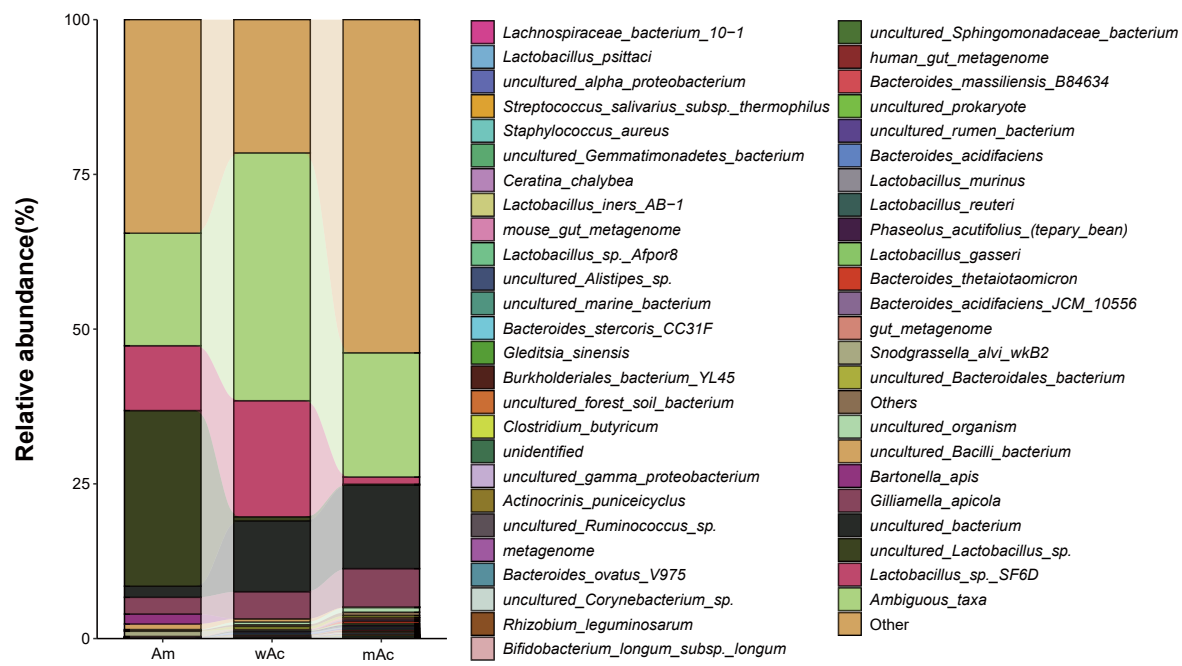

Supplement: S1 Fig — Relative abundance (%) of bacterial taxa in the three groups (Am = Apis mellifera; wAc = wild A. cerana; mAc = managed A. cerana). Panels show taxonomic distributions at four ranks: (A) class, (B) order, (C) family, and (D) species. Bars represent the mean relative abundance for each group, and low-abundance taxa are pooled as “Other.” Relative abundance (%) based on total effective sequence readings. Each group is represented by a single pooled sample (n = 1); consequently, these results are descriptive and do not permit statistical inference of within-group variation. (PDF) [file pone.0339926.s001.pdf]

**A**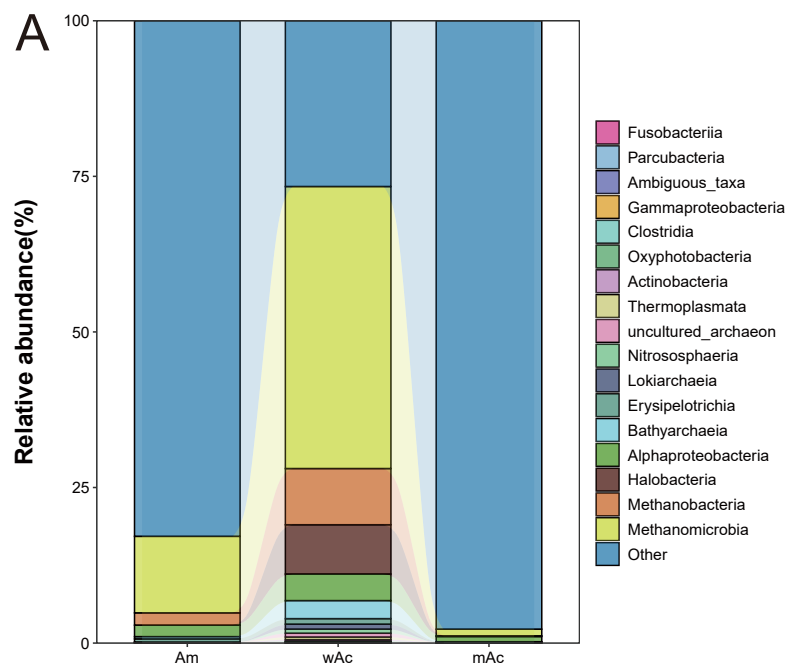**B**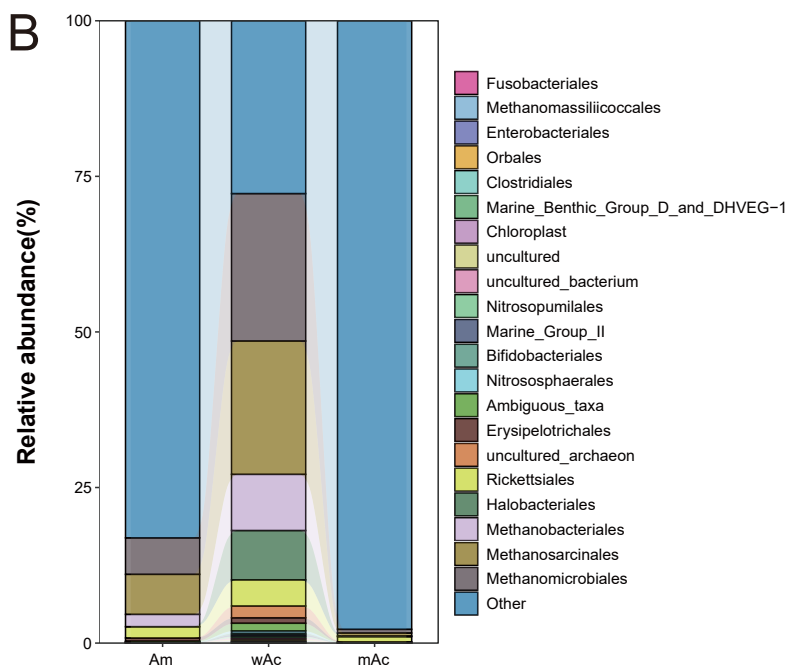**C**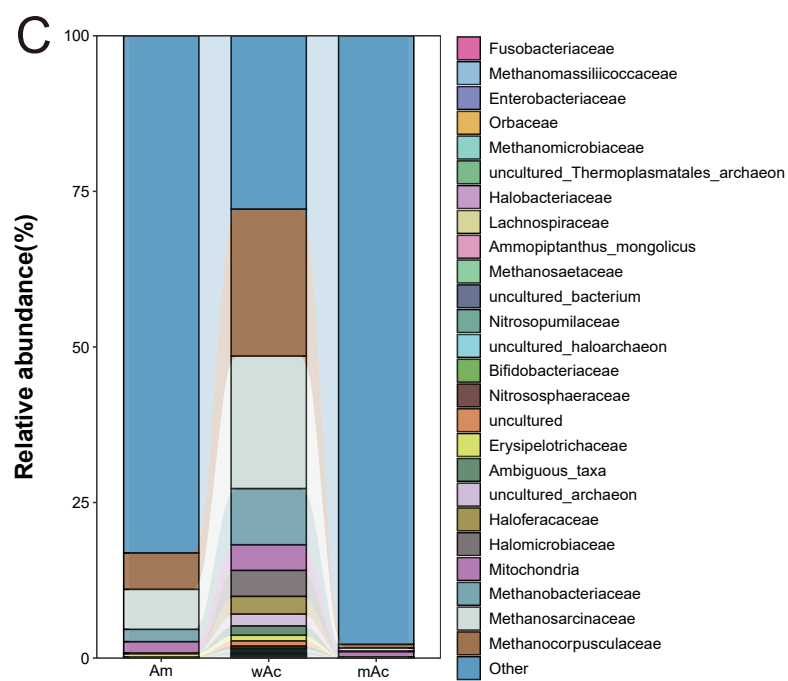**D**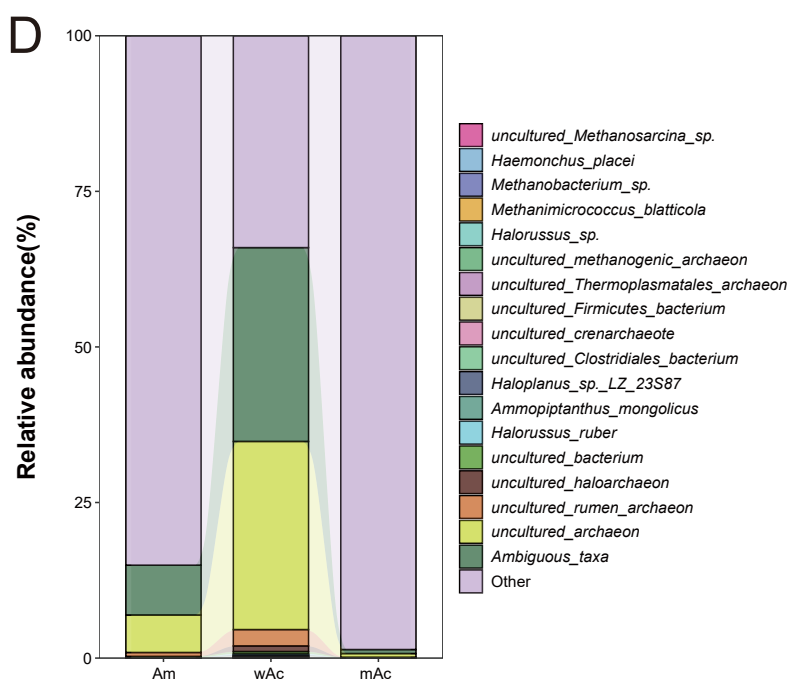

Supplement: S2 Fig — Relative abundance (%) of archaeal taxa in the three groups (Am = A. mellifera; wAc = wild A. cerana; mAc = managed A. cerana). Panels show taxonomic distributions at four ranks: (A) class, (B) order, (C) family, and (D) species. Bars represent the mean relative abundance for each group, and low-abundance taxa are pooled as “Other.” Relative abundance (%) based on total effective sequence readings. Each group is represented by a single pooled sample (n = 1); consequently, these results are descriptive and do not permit statistical inference of within-group variation. (PDF) [file pone.0339926.s002.pdf]

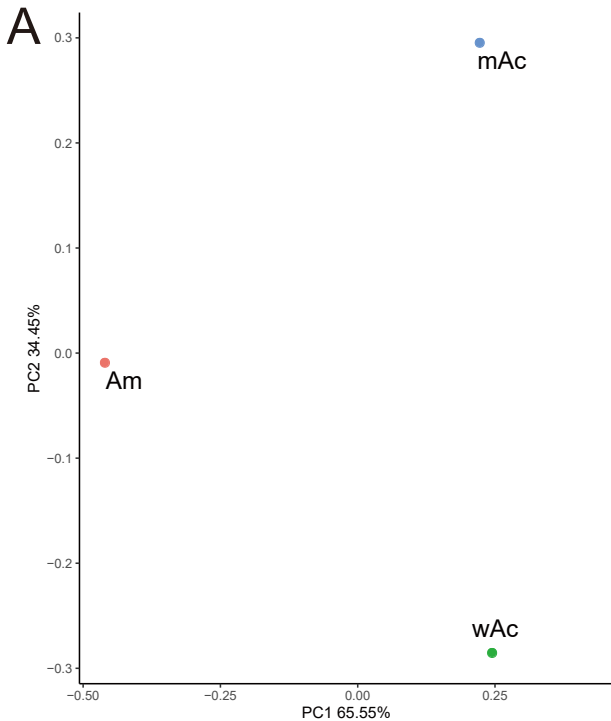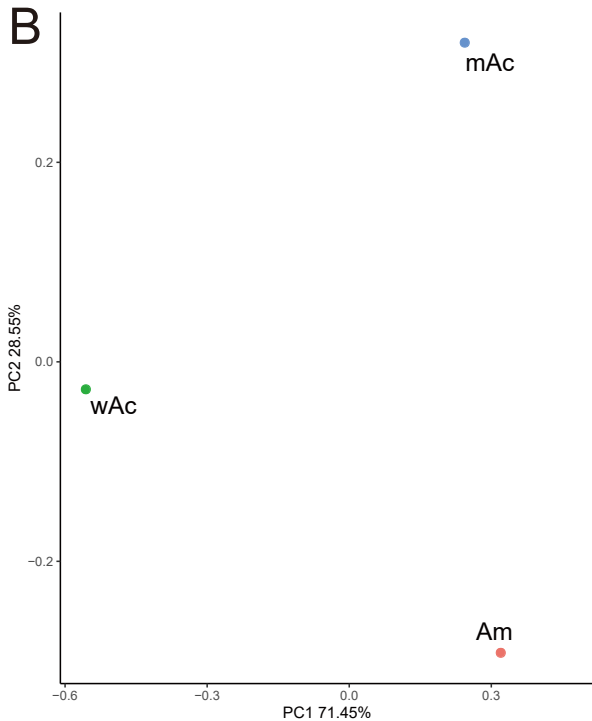

Supplement: S3 Fig — Principal-coordinate analysis (PCA) plots of 16S rRNA gene-based community composition for (A) Bacteria and (B) Archaea. Each point represents one pooled sample per group (Am = A. mellifera; wAc = wild A. cerana; mAc = managed A. cerana), and spatial separation among points reflects compositional dissimilarity among groups. Only used to visually illustrate the overall compositional differences among the three groups; not intended for statistical inference. (PDF) [file pone.0339926.s003.pdf]

A

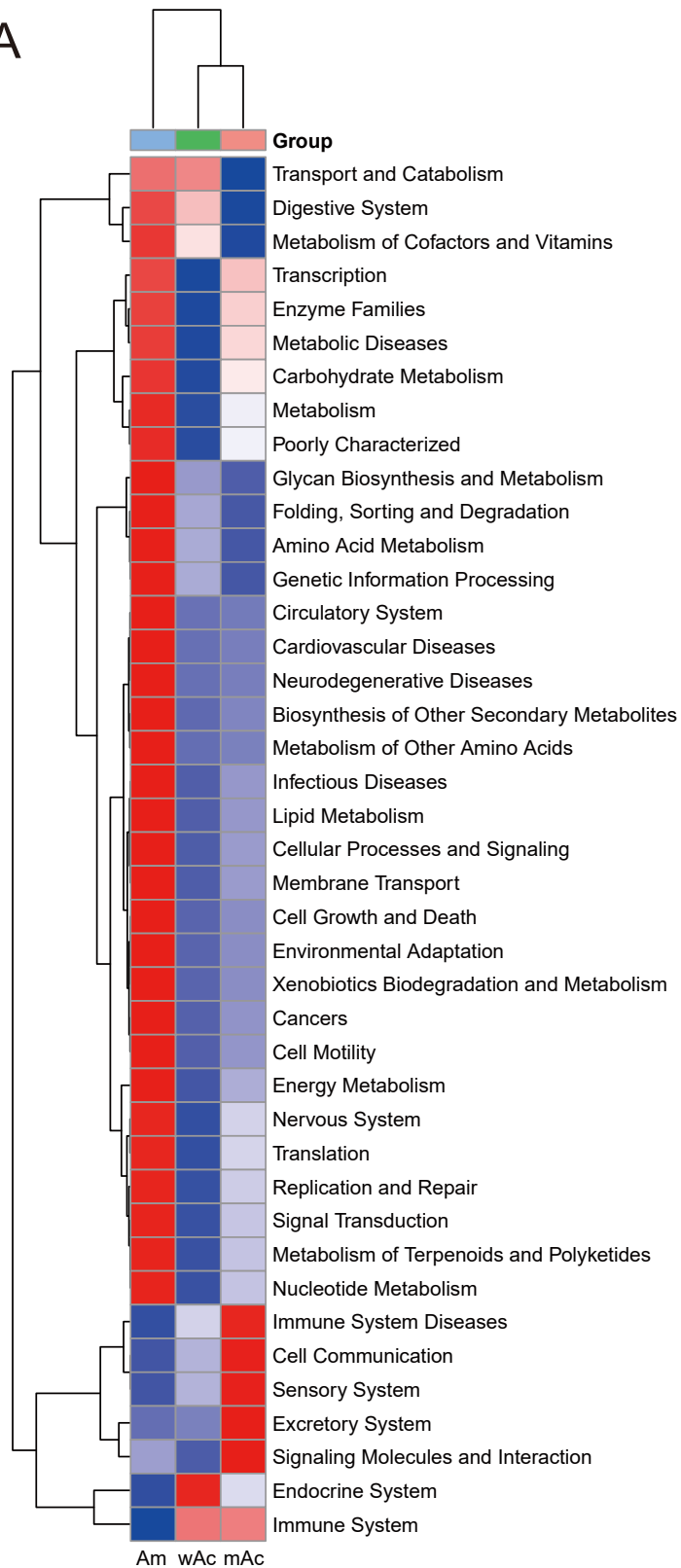

B

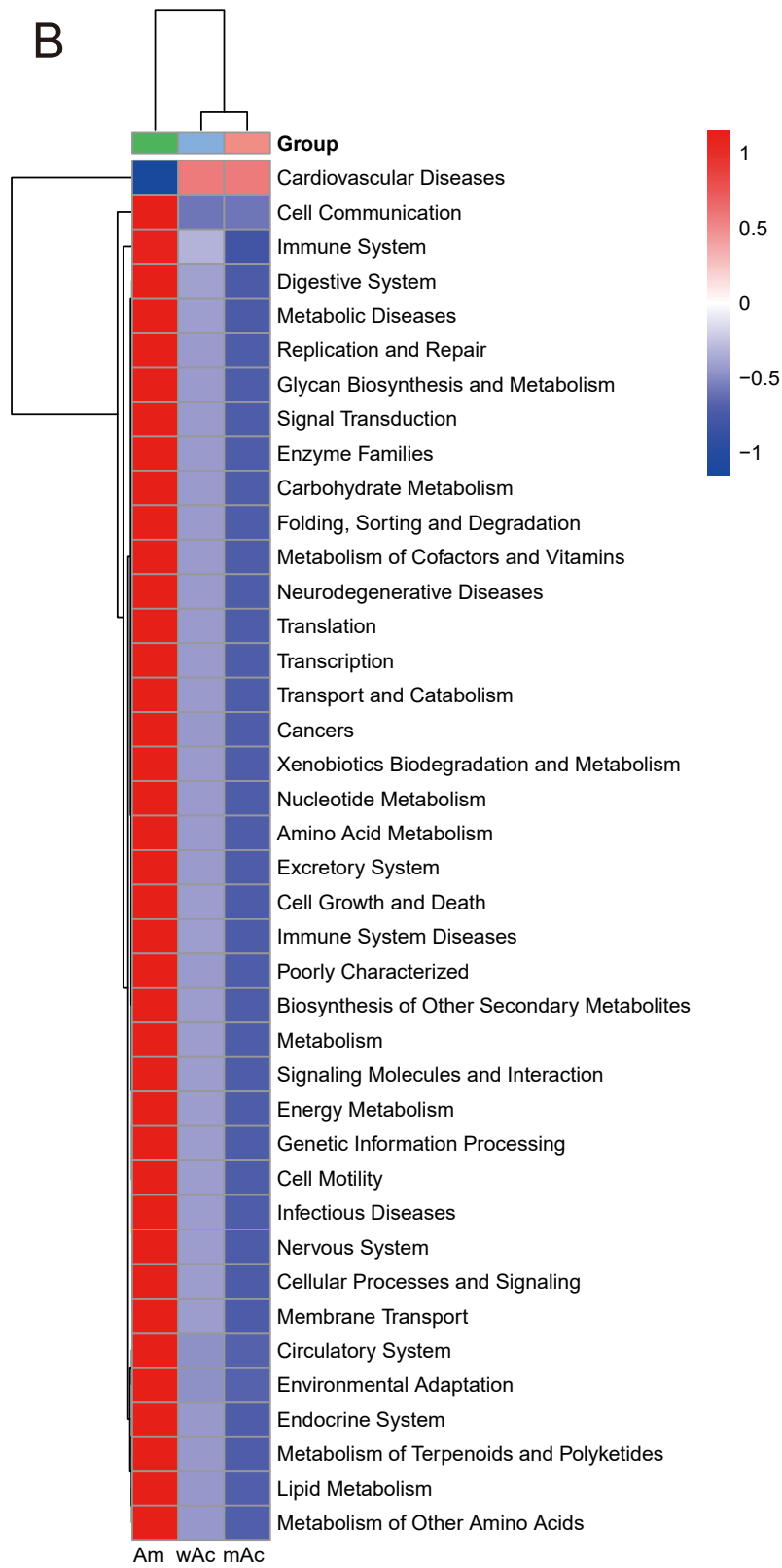

Supplement: S4 Fig — Rows correspond to KEGG Level 2 functional categories, and columns correspond to the three sample groups. Color intensity indicates the normalized relative abundance of each predicted functional category. (PDF) [file pone.0339926.s004.pdf]
