# Supplementary material for: Bacterial-archaeal co-occurrence in honey bee gut microbiomes across host species and management regimes
Source: PLoS One. 2026 Jan 21;21(1):e0339926. doi: 10.1371/journal.pone.0339926 (PMC12822932; doi:10.1371/journal.pone.0339926)
Supplement: Attachment — Submitted filename: PONE_D_25_49948.Miray.docx [file pone.0339926.s005.docx]

# Page-by-page Annotations

**[1] Page 1 – Long sentence** (≈79 words)

Excerpt: “PLOS ONE
Bacterial–Archaeal Co-occurrence in Honey Bee Gut Microbiomes across Host
Species and Management Regimes
--Manuscript Draft--

Manuscript Number: PONE-D-25-49948
Article Type: Research Article
Full Title: Bacterial–Archaeal Co...”

Suggested edit/action: PLOS ONE Bacterial–Archaeal Co-occurrence in Honey Bee Gut Microbiomes across Host Species and Management Regimes --Manuscript Draft-- Manuscript Number: PONE-D-25-49948 Article Type: Research Article Full Title: Bacterial–Archaeal Co-occurrence in Honey Bee Gut Microbiomes across Host Species and Management Regimes Short Title: Bacteria–Araea Co-occurrence in Bee Guts Corresponding Author: Jia-Li Chang Leshan Normal University CHINA Keywords: Apis mellifera; Apis cerana; Gut microbiome; Overwintering; Archaea Abstract: Honeybees are key pollinators, and their overwintering period represents a critical bottleneck for colony survival.

**[2] Page 1 – Long sentence** (≈35 words)

Excerpt: “Order of Authors: Jian-Ping Ying
Yang-Feng Zou
Tao Jiang
Jia-Li Chang
Additional Information:
Question Response
Financial Disclosure
Enter a financial disclosure statement that
describes the sources of funding for the
work included in this ...”

Suggested edit/action: Order of Authors: Jian-Ping Ying Yang-Feng Zou Tao Jiang Jia-Li Chang Additional Information: Question Response Financial Disclosure Enter a financial disclosure statement that describes the sources of funding for the work included in this submission.

**[3] Page 1 – Strong claim wording**

Excerpt: “Notably,
wAc exhibited a significant enrichment of methanogenic archaea (e.g.,
Methanocorpusculaceae and Methanosarcinaceae), a pattern consistent with
bacterial–archaeal metabolic coupling, which may improve fermentation
thermodynamics and...”

Suggested edit/action: Notably,
wAc exhibited a suggests|indicates (provide p-value and 95% CI) enrichment of methanogenic archaea (e.g.,
Methanocorpusculaceae and Methanosarcinaceae), a pattern consistent with
bacterial–archaeal metabolic coupling, which may improve fermentation
thermodynamics and energy extraction during winter’s resource-limited conditions.

**[4] Page 2 – Long sentence** (≈65 words)

Excerpt: “Funded studies
Enter a statement with the following details:
Initials of the authors who received each
award•
Grant numbers awarded to each author•
The full name of each funder•
URL of each funder website•
Did the sponsors or funders play a...”

Suggested edit/action: Funded studies Enter a statement with the following details: Initials of the authors who received each award• Grant numbers awarded to each author• The full name of each funder• URL of each funder website• Did the sponsors or funders play any role in the study design, data collection and analysis, decision to publish, or preparation of the manuscript?• Did you receive funding for this work?

**[5] Page 2 – Long sentence** (≈83 words)

Excerpt: “Funded studies
Enter a statement with the following details:
Initials of the authors who received each
award•
Grant numbers awarded to each author•
The full name of each funder•
URL of each funder website•
Did the sponsors or funders play a...”

Suggested edit/action: Funded studies Enter a statement with the following details: Initials of the authors who received each award• Grant numbers awarded to each author• The full name of each funder• URL of each funder website• Did the sponsors or funders play any role in the study design, data collection and analysis, decision to publish, or preparation of the manuscript?• Did you receive funding for this work?"National Natural Science Foundation of China (No. 52200056) Powered by Editorial Manager® and ProduXion Manager® from Aries Systems Corporation

**[6] Page 3 – Long sentence** (≈45 words)

Excerpt: “Please select the country of your main
research funder (please select carefully
as in some cases this is used in fee
calculation).
 as follow-up to "Financial Disclosure
Enter a financial disclosure statement that
describes the sources of f...”

Suggested edit/action: Please select the country of your main research funder (please select carefully as in some cases this is used in fee calculation). as follow-up to "Financial Disclosure Enter a financial disclosure statement that describes the sources of funding for the work included in this submission.

**[7] Page 3 – Long sentence** (≈82 words)

Excerpt: “Funded studies
Enter a statement with the following details:
Initials of the authors who received each
award•
Grant numbers awarded to each author•
The full name of each funder•
URL of each funder website•
Did the sponsors or funders play a...”

Suggested edit/action: Funded studies Enter a statement with the following details: Initials of the authors who received each award• Grant numbers awarded to each author• The full name of each funder• URL of each funder website• Did the sponsors or funders play any role in the study design, data collection and analysis, decision to publish, or preparation of the manuscript?• Did you receive funding for this work?"CHINA - CN Competing Interests Use the instructions below to enter a competing interest statement for this submission.

**[8] Page 4 – Long sentence** (≈44 words)

Excerpt: “Authors with competing interests
Enter competing interest details beginning
with this statement:
I have read the journal's policy and the
authors of this manuscript have the following
competing interests: [insert competing
interests here...”

Suggested edit/action: Authors with competing interests Enter competing interest details beginning with this statement: I have read the journal's policy and the authors of this manuscript have the following competing interests: [insert competing interests here] * typeset Ethics Statement Enter an ethics statement for this submission.

**[9] Page 4 – Long sentence** (≈35 words)

Excerpt: “This statement is required if
the study involved:
Human participants•
Human specimens or tissue•
Vertebrate animals or cephalopods•
Vertebrate embryos or tissues•
Field research•
Write "N/A" if the submission does not
require an ethics stat...”

Suggested edit/action: This statement is required if the study involved: Human participants• Human specimens or tissue• Vertebrate animals or cephalopods• Vertebrate embryos or tissues• Field research• Write "N/A" if the submission does not require an ethics statement.

**[10] Page 4 – Policy marker: ethics (mention found)**

Excerpt: “sources listed in your Funding Information later in the submission form are also declared in your Financial Disclosure statement. View published research articles from PLOS ONE for specific examples....”

Suggested edit/action: Verify that PLOS ONE-compliant details (DOIs/accessions, permit numbers, explicit statements) are fully provided.

**[11] Page 5 – Long sentence** (≈213 words)

Excerpt: “Format for specific study types
Human Subject Research (involving human
participants and/or tissue)
Give the name of the institutional review
board or ethics committee that approved the
study•
Include the approval number and/or a
statement ...”

Suggested edit/action: Format for specific study types Human Subject Research (involving human participants and/or tissue) Give the name of the institutional review board or ethics committee that approved the study• Include the approval number and/or a statement indicating approval of this research• Indicate the form of consent obtained (written/oral) or the reason that consent was not obtained (e.g. the data were analyzed anonymously)• Animal Research (involving vertebrate animals, embryos or tissues) Provide the name of the Institutional Animal Care and Use Committee (IACUC) or other relevant ethics board that reviewed the study protocol, and indicate whether they approved this research or granted a formal waiver of ethical approval• Include an approval number if one was obtained• If the study involved non-human primates , add additional details about animal welfare and steps taken to ameliorate suffering• If anesthesia, euthanasia, or any kind of animal sacrifice is part of the study, include briefly which substances and/or methods were applied• Field Research Include the following details if this study involves the collection of plant, animal, or other materials from a natural setting: Field permit number• Name of the institution or relevant body that granted permission• Data Availability Authors are required to make all data underlying the findings described fully available, without restriction, and from the time of publication.

**[12] Page 5 – Policy marker: data_availability (mention found)**

Excerpt: “Format for specific study types Human Subject Research (involving human participants and/or tissue) Give the name of the institutional review board or ethics committee that approved the study• Include...”

Suggested edit/action: Verify that PLOS ONE-compliant details (DOIs/accessions, permit numbers, explicit statements) are fully provided.

**[13] Page 5 – Policy marker: ethics (mention found)**

Excerpt: “Format for specific study types Human Subject Research (involving human participants and/or tissue) Give the name of the institutional review board or ethics committee that approved the study• Include...”

Suggested edit/action: Verify that PLOS ONE-compliant details (DOIs/accessions, permit numbers, explicit statements) are fully provided.

**[14] Page 6 – Long sentence** (≈67 words)

Excerpt: “For example: All XXX files
are available from the XXX database
(accession number(s) XXX, XXX.). •
If the data are all contained within the
manuscript and/or Supporting
Information files, enter the following:
All relevant data are within t...”

Suggested edit/action: For example: All XXX files are available from the XXX database (accession number(s) XXX, XXX.). • If the data are all contained within the manuscript and/or Supporting Information files, enter the following: All relevant data are within the manuscript and its Supporting Information files. • If neither of these applies but you are able to provide details of access elsewhere, with or without limitations, please do so.

**[15] Page 6 – Policy marker: data_availability (mention found)**

Excerpt: “A Data Availability Statement describing where the data can be found is required at submission. Your answers to this question constitute the Data Availability Statement and will be published in the ar...”

Suggested edit/action: Verify that PLOS ONE-compliant details (DOIs/accessions, permit numbers, explicit statements) are fully provided.

**[16] Page 6 – Policy marker: ethics (mention found)**

Excerpt: “A Data Availability Statement describing where the data can be found is required at submission. Your answers to this question constitute the Data Availability Statement and will be published in the ar...”

Suggested edit/action: Verify that PLOS ONE-compliant details (DOIs/accessions, permit numbers, explicit statements) are fully provided.

**[17] Page 6 – Policy marker: code_repo (mention found)**

Excerpt: “A Data Availability Statement describing where the data can be found is required at submission. Your answers to this question constitute the Data Availability Statement and will be published in the ar...”

Suggested edit/action: Verify that PLOS ONE-compliant details (DOIs/accessions, permit numbers, explicit statements) are fully provided.

**[18] Page 7 – Long sentence** (≈55 words)

Excerpt: “This text is appropriate if the data are
owned by a third party and authors do
not have permission to share the data.•
* typeset
Additional data availability information: Tick here if the URLs/accession numbers/DOIs will be available only a...”

Suggested edit/action: This text is appropriate if the data are owned by a third party and authors do not have permission to share the data.• * typeset Additional data availability information: Tick here if the URLs/accession numbers/DOIs will be available only after acceptance of the manuscript for publication so that we can ensure their inclusion before publication.

**[19] Page 7 – Policy marker: data_availability (mention found)**

Excerpt: “and contact information or URL). This text is appropriate if the data are owned by a third party and authors do not have permission to share the data.• * typeset Additional data availability informat...”

Suggested edit/action: Verify that PLOS ONE-compliant details (DOIs/accessions, permit numbers, explicit statements) are fully provided.

**[20] Page 7 – Policy marker: ethics (mention found)**

Excerpt: “and contact information or URL). This text is appropriate if the data are owned by a third party and authors do not have permission to share the data.• * typeset Additional data availability informat...”

Suggested edit/action: Verify that PLOS ONE-compliant details (DOIs/accessions, permit numbers, explicit statements) are fully provided.

**[21] Page 7 – Policy marker: code_repo (mention found)**

Excerpt: “and contact information or URL). This text is appropriate if the data are owned by a third party and authors do not have permission to share the data.• * typeset Additional data availability informat...”

Suggested edit/action: Verify that PLOS ONE-compliant details (DOIs/accessions, permit numbers, explicit statements) are fully provided.

**[22] Page 8 – Long sentence** (≈52 words)

Excerpt: “Bacterial –Archaeal Co -occurrence in Honey Bee Gut Microbiomes
across Host Species and Management Regimes
Jian-Ping Ying1†, Yang -Feng Zou1†, Tao Jiang1,*, Jia-Li Chang1,*
1Department of New Energy Materials and Chemistry, Leshan Norma...”

Suggested edit/action: Bacterial –Archaeal Co -occurrence in Honey Bee Gut Microbiomes across Host Species and Management Regimes Jian-Ping Ying1†, Yang -Feng Zou1†, Tao Jiang1,*, Jia-Li Chang1,* 1Department of New Energy Materials and Chemistry, Leshan Normal University, Leshan 61 4004, China †Contributed equally to this work *Correspondence: jialiyangfeng@163.com or composting@163.com Manuscript Click here to access/download;Manuscript;manuscript.doc

**[23] Page 9 – Long sentence** (≈36 words)

Excerpt: “We investigated how hos t species and husbandry 3
practices influence the composition of gut bacterial and archaeal communities of 4
overwintering honeybees, as well as the potential functional consequences for energy 5
metabolism and re...”

Suggested edit/action: We investigated how hos t species and husbandry 3 practices influence the composition of gut bacterial and archaeal communities of 4 overwintering honeybees, as well as the potential functional consequences for energy 5 metabolism and resilience.

**[24] Page 9 – Long sentence** (≈40 words)

Excerpt: “Notably, 8
wAc exhibited a significant enrichment of methanogenic archaea (e.g., 9
Methanocorpu sculaceae and Methanosarcinaceae ), a pattern consistent with 10
bacterial –archaeal metabolic coupling, which may improve fermentation 11
...”

Suggested edit/action: Notably, 8 wAc exhibited a significant enrichment of methanogenic archaea (e.g., 9 Methanocorpu sculaceae and Methanosarcinaceae ), a pattern consistent with 10 bacterial –archaeal metabolic coupling, which may improve fermentation 11 thermodynamics and energy extraction during winter’s resource -limited conditions.

**[25] Page 9 – Long sentence** (≈39 words)

Excerpt: “In mAc, we observed a relative enric hment of 14
carbohydrate metabolism and replication/repair pathways, suggesting that artificial 15
feeding and high -density management may steer the micro biome toward a high - 16
throughput but lowe...”

Suggested edit/action: In mAc, we observed a relative enric hment of 14 carbohydrate metabolism and replication/repair pathways, suggesting that artificial 15 feeding and high -density management may steer the micro biome toward a high - 16 throughput but lower -redundancy configuration.

**[26] Page 9 – Strong claim wording**

Excerpt: “Notably, 8
wAc exhibited a significant enrichment of methanogenic archaea (e.g., 9
Methanocorpu sculaceae and Methanosarcinaceae ), a pattern consistent with 10
bacterial –archaeal metabolic coupling, which may improve fermentation 11
...”

Suggested edit/action: Notably, 8
wAc exhibited a suggests|indicates (provide p-value and 95% CI) enrichment of methanogenic archaea (e.g., 9
Methanocorpu sculaceae and Methanosarcinaceae ), a pattern consistent with 10
bacterial –archaeal metabolic coupling, which may improve fermentation 11
thermodynamics and energy extraction during winter’s resource -limited conditions.

**[27] Page 10 – Long sentence** (≈41 words)

Excerpt: “Consequently, two bee taxa have become 31
focal points of both applied and basic research: the western honeybee ( Apis mellifera , 32
hereafter Am) and the eastern honeybee complex (principally Apis cerana and its 33
closely related lin...”

Suggested edit/action: Consequently, two bee taxa have become 31 focal points of both applied and basic research: the western honeybee ( Apis mellifera , 32 hereafter Am) and the eastern honeybee complex (principally Apis cerana and its 33 closely related lineages, hereafter Ac).

**[28] Page 10 – Long sentence** (≈45 words)

Excerpt: “A. mellifera has been 36
widely transported and intensively managed on a global sca le for commercial 37
pollination [3], with large colony sizes, frequent translocation s, monocultural foraging 38
landscapes, and recurrent chemical in...”

Suggested edit/action: A. mellifera has been 36 widely transported and intensively managed on a global sca le for commercial 37 pollination [3], with large colony sizes, frequent translocation s, monocultural foraging 38 landscapes, and recurrent chemical interventions (e.g., acaricides and antibiotics) 39 characterizing many Am operations [4-6].

**[29] Page 11 – Long sentence** (≈37 words)

Excerpt: “may influence physiology, disease susceptibility, and resilience to seasonal stressors. 45
Overwintering is one of the most critical phases in the annual cycle of honey bee 46
colonies, and represents a pronounced bottleneck for populati...”

Suggested edit/action: may influence physiology, disease susceptibility, and resilience to seasonal stressors. 45 Overwintering is one of the most critical phases in the annual cycle of honey bee 46 colonies, and represents a pronounced bottleneck for population persistence [8].

**[30] Page 11 – Long sentence** (≈37 words)

Excerpt: “Behavioral thermoregulation — 50
most notably the formation of compact overwintering clusters and the generation of 51
metabolic heat —enables colonies to maintain a warm core even when ambient 52
temperatures fall belo w 10 °C [9].”

Suggested edit/action: Behavioral thermoregulation — 50 most notably the formation of compact overwintering clusters and the generation of 51 metabolic heat —enables colonies to maintain a warm core even when ambient 52 temperatures fall belo w 10 °C [9].

**[31] Page 11 – Long sentence** (≈75 words)

Excerpt: “These strategies, however, are energetically 53
expensive and require substantial autumnal accumul ation of nectar - and pollen - 54
derived reserves; colonies that fail to secure adequate stores or that experience 55
disruptions in fora...”

Suggested edit/action: These strategies, however, are energetically 53 expensive and require substantial autumnal accumul ation of nectar - and pollen - 54 derived reserves; colonies that fail to secure adequate stores or that experience 55 disruptions in foraging or health are at an elevated risk of overwinter loss [10]. 56 Common beekeeper interventions (e.g., supplemental feeding, insulation, and Varroa 57 control) can mitigate some risks, but they may also alter nutritional inputs and 58 microbial exposures [6].

**[32] Page 11 – Long sentence** (≈35 words)

Excerpt: “Notably, interspecific differences are important; some eastern 59
honeybee populations sustain foraging and activity at lower tempera tures and exhibit 60
physiological traits that confer greater cold tolerance than many western stocks ...”

Suggested edit/action: Notably, interspecific differences are important; some eastern 59 honeybee populations sustain foraging and activity at lower tempera tures and exhibit 60 physiological traits that confer greater cold tolerance than many western stocks [11, 61 12].

**[33] Page 11 – Long sentence** (≈38 words)

Excerpt: “These differences may interact with hos t-associated microbiota to influence 62
overwintering outcomes . 63
The gut microbiome is increasingly recognized as a central regulator of insect 64
health and a mediator of ecological and physiol...”

Suggested edit/action: These differences may interact with hos t-associated microbiota to influence 62 overwintering outcomes . 63 The gut microbiome is increasingly recognized as a central regulator of insect 64 health and a mediator of ecological and physiological plasticity [12].

**[34] Page 11 – Policy marker: code_repo (mention found)**

Excerpt: “may influence physiology, disease susceptibility, and resilience to seasonal stressors. 45 Overwintering is one of the most critical phases in the annual cycle of honey bee 46 colonies, and represe...”

Suggested edit/action: Verify that PLOS ONE-compliant details (DOIs/accessions, permit numbers, explicit statements) are fully provided.

**[35] Page 12 – Long sentence** (≈48 words)

Excerpt: “For example, fermentative bacteria generate short -chain fatty acids [17] 71
and other metabolites [18, 19] that can be utilized by the host or by other microbial 72
partners, whereas certain core taxa competitively exclud e opportunis...”

Suggested edit/action: For example, fermentative bacteria generate short -chain fatty acids [17] 71 and other metabolites [18, 19] that can be utilized by the host or by other microbial 72 partners, whereas certain core taxa competitively exclud e opportunistic pathogens that 73 proliferate when the host defenses are compromised [20].

**[36] Page 13 – Long sentence** (≈76 words)

Excerpt: “As microbiome composition mediates nutrient processing, 89
detoxification, and immune modulation, structural differences in microbiome between 90
wild an d managed bees —particularly during overwintering —may translate into 91
measurable...”

Suggested edit/action: As microbiome composition mediates nutrient processing, 89 detoxification, and immune modulation, structural differences in microbiome between 90 wild an d managed bees —particularly during overwintering —may translate into 91 measurable differences in energy balance , pathogen resistance, and ultimately survival . 92 Motivated by these ecological and management -relevant considerations, the 93 present study compared the gut bacterial and archaeal communities of overwintering 94 A. mellifera (Am), wild A. cerana (wAc), and managed A. cerana (mAc).

**[37] Page 13 – Long sentence** (≈49 words)

Excerpt: “Using 16S 95
rRNA amplicon sequencing, we quantified alpha - and beta -diversity, characterized 96
taxonomic composition across multiple ranks, and applied marker -based functional 97
prediction (Kyoto Encyclopedia of Genes and Genomes [...”

Suggested edit/action: Using 16S 95 rRNA amplicon sequencing, we quantified alpha - and beta -diversity, characterized 96 taxonomic composition across multiple ranks, and applied marker -based functional 97 prediction (Kyoto Encyclopedia of Genes and Genomes [KEGG] classi fications) to 98 infer potential metabolic capacities associated with each host –management 99 combination.

**[38] Page 13 – Long sentence** (≈50 words)

Excerpt: “Our objectives were to determine (i) whether ho st species and 100
management status predictably structure microbial diversity during overwintering, (ii) 101
which bacterial and archaeal taxa distinguish wild from managed eastern and west...”

Suggested edit/action: Our objectives were to determine (i) whether ho st species and 100 management status predictably structure microbial diversity during overwintering, (ii) 101 which bacterial and archaeal taxa distinguish wild from managed eastern and western 102 bees, and (iii) which inferred functional pathways may plausibly suppo rt overwinter 103 metabolic demands.

**[39] Page 13 – Long sentence** (≈43 words)

Excerpt: “By integrating taxonomic and functional perspectives, we aimed 104
to identify candidate microbial processes that could contribute to overwinter resilience 105
and inform microbiome -aware strategies for supporting colony health, in cludi...”

Suggested edit/action: By integrating taxonomic and functional perspectives, we aimed 104 to identify candidate microbial processes that could contribute to overwinter resilience 105 and inform microbiome -aware strategies for supporting colony health, in cluding 106 targeted dietary interventions and microbiome restoration approaches. 107 108 2.

**[40] Page 14 – Long sentence** (≈38 words)

Excerpt: “The collected bee 116
samples were placed in sealed centrifuge tubes and stored at -20°C. 117
2.2 Extraction of sample DNA 118
The intestinal contents collecti on method was slightly modified based on the previous 119
report [24].”

Suggested edit/action: The collected bee 116 samples were placed in sealed centrifuge tubes and stored at -20°C. 117 2.2 Extraction of sample DNA 118 The intestinal contents collecti on method was slightly modified based on the previous 119 report [24].

**[41] Page 14 – Long sentence** (≈134 words)

Excerpt: “The bee samples were placed on 121
sterile plates in a sterile ultra -clean be nch; Using forceps, the bee intestines were 122
removed and placed in centrifuge tubes containing phosphate -buffered saline; using a 123
sterile syringe, the...”

Suggested edit/action: The bee samples were placed on 121 sterile plates in a sterile ultra -clean be nch; Using forceps, the bee intestines were 122 removed and placed in centrifuge tubes containing phosphate -buffered saline; using a 123 sterile syringe, the intestinal wall was punctured, and the centrifuge tubes were shaken 124 for 15 minutes to ensure complete rele ase of intestinal contents; using a sterile needle, 125 the intestinal wall was removed, and the remaining bacte rial suspension was used for 126 nucleic acid DNA extraction following the protocol of the FastDNA Spin Kit for 127 Feces kit manufacturer's instructions. 128 2.3 PCR and sequencing for 16S rRNA 129 The V3 -V4 hypervariable region of the bacterial 16S rRNA gene was amplified using 130 primer pair 338F (5′ -ACTCCTACGGGAGGCAGCAG -3') and 806R (5′ - 131

**[42] Page 15 – Long sentence** (≈38 words)

Excerpt: “The V4 region of the 16S rRNA gene of 132
archaea was amplified using primers 524F (5' -TGYCAGCCGCCGCGGAA -3') and 133
958R (5' -YCCGGCGTTGA VTCCAATT -3') on an ABI GeneAmp 9700 PCR thermal 134
cycler (ABI, USA) [25].”

Suggested edit/action: The V4 region of the 16S rRNA gene of 132 archaea was amplified using primers 524F (5' -TGYCAGCCGCCGCGGAA -3') and 133 958R (5' -YCCGGCGTTGA VTCCAATT -3') on an ABI GeneAmp 9700 PCR thermal 134 cycler (ABI, USA) [25].

**[43] Page 15 – Long sentence** (≈44 words)

Excerpt: “The PCR reaction mixture included: 4 μL 5× Fast Pfu buffer, 135
2 μL 2.5 mM dNTPs, 0.8 μL of each primer (5 μM concentration), 0.4 μL Fast Pfu 136
polymerase, 10 ng template DNA, and ddH₂O to a final volume of 20 μL.”

Suggested edit/action: The PCR reaction mixture included: 4 μL 5× Fast Pfu buffer, 135 2 μL 2.5 mM dNTPs, 0.8 μL of each primer (5 μM concentration), 0.4 μL Fast Pfu 136 polymerase, 10 ng template DNA, and ddH₂O to a final volume of 20 μL.

**[44] Page 15 – Long sentence** (≈52 words)

Excerpt: “PCR 137
amplification cycle conditions were as follows: initial denaturation at 95°C for 5 138
minutes, followed by 34 cycles, each cycle including denaturatio n at 94°C for 30 139
seconds, annealing at 57°C for 30 seconds, extension at ...”

Suggested edit/action: PCR 137 amplification cycle conditions were as follows: initial denaturation at 95°C for 5 138 minutes, followed by 34 cycles, each cycle including denaturatio n at 94°C for 30 139 seconds, annealing at 57°C for 30 seconds, extension at 72°C for 60 seconds, and 140 final extension at 72°C for 10 minutes.

**[45] Page 15 – Long sentence** (≈42 words)

Excerpt: “(Shanghai, China) according to standard procedures. 148
2.4 Data processing and bioinfor matics analysis 149
Raw FASTQ files were filtered to remove reads with single nucleotide repeat lengths 150
≥10 bp, no overlap (overlap = 15 bp), ...”

Suggested edit/action: (Shanghai, China) according to standard procedures. 148 2.4 Data processing and bioinfor matics analysis 149 Raw FASTQ files were filtered to remove reads with single nucleotide repeat lengths 150 ≥10 bp, no overlap (overlap = 15 bp), and reads containing N bases.

**[46] Page 15 – Policy marker: code_repo (mention found)**

Excerpt: “GGACTACHVGGGTWTCTAAT -3') [25]. The V4 region of the 16S rRNA gene of 132 archaea was amplified using primers 524F (5' -TGYCAGCCGCCGCGGAA -3') and 133 958R (5' -YCCGGCGTTGA VTCCAATT -3') on an ABI...”

Suggested edit/action: Verify that PLOS ONE-compliant details (DOIs/accessions, permit numbers, explicit statements) are fully provided.

**[47] Page 16 – Long sentence** (≈36 words)

Excerpt: “were removed based on a minimum length of 15 bp and a mismatch rate of 0.1. 154
Subsequently, tags were cluste red into operational taxonomic units (OTUs) using the 155
USEARCH software (97% sequence homology) [26].”

Suggested edit/action: were removed based on a minimum length of 15 bp and a mismatch rate of 0.1. 154 Subsequently, tags were cluste red into operational taxonomic units (OTUs) using the 155 USEARCH software (97% sequence homology) [26].

**[48] Page 16 – Long sentence** (≈36 words)

Excerpt: “Finally, 157
representative OTU sequences were annotated with species -level taxonomic 158
annotations using the RDP classifier (v2.2) based on the Silva 138.1 SSU reference 159
database (release date: August 2020), with a confidence lev...”

Suggested edit/action: Finally, 157 representative OTU sequences were annotated with species -level taxonomic 158 annotations using the RDP classifier (v2.2) based on the Silva 138.1 SSU reference 159 database (release date: August 2020), with a confidence level ≥0.6.

**[49] Page 16 – Long sentence** (≈56 words)

Excerpt: “For α diversity analysis, OTU richness, Shannon index, Simpson 162
index, Chao index, ACE index, and coverage index were calculated using mothur and 163
plotted using R. β diversity was assessed using cluster analysis (PCA and NMDS) in 1...”

Suggested edit/action: For α diversity analysis, OTU richness, Shannon index, Simpson 162 index, Chao index, ACE index, and coverage index were calculated using mothur and 163 plotted using R. β diversity was assessed using cluster analysis (PCA and NMDS) in 164 QIIME2 [28], and statistical testing was performed using ANOSIM analysis with the 165 Euclidean algorithm. 166 3.

**[50] Page 17 – Long sentence** (≈130 words)

Excerpt: “In contrast, Fig 1B shows that the overall archaeal 183
diversity was comparatively low, with only 24 core OTUs shared among the 3 groups. 184
Unique archaeal OTUs included 96 in Am, 239 in wAc, and 36 in mAc , while 185
pairwise overlap...”

Suggested edit/action: In contrast, Fig 1B shows that the overall archaeal 183 diversity was comparatively low, with only 24 core OTUs shared among the 3 groups. 184 Unique archaeal OTUs included 96 in Am, 239 in wAc, and 36 in mAc , while 185 pairwise overlaps were 21 for Am –wAc, 93 for Am –mAc, and 36 for wAc –mAc. 186 3.2 Composition of gut microbiota in Am, wAc, and mAc during the wintering period 187 Across samples, the intestinal microbiome was numerically dominated by a small 188 number of high -abundance taxa, although the bacterial and archaeal fractions 189 exhibited distinct compositional patterns among groups. 190 At the phylum level (Figure 2A), bacterial communities were primarily 191 represented by Firmicutes and Proteobacteria , with markedly differe nt contributions 192 across groups.

**[51] Page 17 – Strong claim wording**

Excerpt: “The OTU count for the Am 176
sample was 827, significantly lower than that of wAc (1,380) and mAc (1,574).”

Suggested edit/action: The OTU count for the Am 176
sample was 827, suggests|indicates (provide p-value and 95% CI) lower than that of wAc (1,380) and mAc (1,574).

**[52] Page 18 – Long sentence** (≈58 words)

Excerpt: “At the order level, Lactobacillales 203
dominated each group (Am, 43.7%; mAc, 52.9%; wAc, 39.0%), whereas orders 204
affiliated with Proteobacteria and Bacteroidetes (e.g., Rhizobiales and Bacteroidales) 205
contributed substan tially to...”

Suggested edit/action: At the order level, Lactobacillales 203 dominated each group (Am, 43.7%; mAc, 52.9%; wAc, 39.0%), whereas orders 204 affiliated with Proteobacteria and Bacteroidetes (e.g., Rhizobiales and Bacteroidales) 205 contributed substan tially to Am and wAc but were comparatively depleted in mAc. 206 Family - (Fig S1C) and genus -level (Fig 2B) analyses revealed pronounced enrichment 207 of lactobacilli.

**[53] Page 18 – Long sentence** (≈44 words)

Excerpt: “Lactobacillaceae (family) and Lactobacillus (genus) were the largest 208
taxonomic components in al l groups, with family -level abundances of 43.7%, 52.8% 209
and 38.9% for Am, mAc, and wAc, respectively, and corresponding genus -level ...”

Suggested edit/action: Lactobacillaceae (family) and Lactobacillus (genus) were the largest 208 taxonomic components in al l groups, with family -level abundances of 43.7%, 52.8% 209 and 38.9% for Am, mAc, and wAc, respectively, and corresponding genus -level 210 abundances of Lactobacillus (Am,43.7%; mAc, 52.8%; wAc, 39.0%).

**[54] Page 18 – Long sentence** (≈83 words)

Excerpt: “Secondary taxa 211
varied by group: Rhizobiaceae (38.6%) were unu sually abundant in Am, whereas wAc 212
contained notable proportions of Orbaceae (20.4%) and Bifidobacteriaceae (14.9%). 213
At the genus level, in addition to Lactobacill...”

Suggested edit/action: Secondary taxa 211 varied by group: Rhizobiaceae (38.6%) were unu sually abundant in Am, whereas wAc 212 contained notable proportions of Orbaceae (20.4%) and Bifidobacteriaceae (14.9%). 213 At the genus level, in addition to Lactobacillus , Gilliamella and Bifidobacterium were 214 detectable as secondary contributors, especially in wAc. 215 Species -level assignments (Fig S1D) for bacteria were constrained by ambiguous 216 or uncultured annotations; for example, in mAc, “Other” and “Ambiguous_taxa” 217 accounted for 53.8% and 20.1%, respectively, whereas in wAc, “Ambiguous_taxa” 218

**[55] Page 19 – Long sentence** (≈50 words)

Excerpt: “These results indicate pervasive Firmicutes 222
dominance largely driven by Lactobacillus , with the Firmicutes/ Lactobacillus 223
enrichment most pronounced in mAc, and larger Proteobacterial and Actinobacterial 224
contributions in A...”

Suggested edit/action: These results indicate pervasive Firmicutes 222 dominance largely driven by Lactobacillus , with the Firmicutes/ Lactobacillus 223 enrichment most pronounced in mAc, and larger Proteobacterial and Actinobacterial 224 contributions in Am and wAc, respectively. 225 The archaeal c omposition differed markedly among the groups and exhibited 226 variable annotation resolution.

**[56] Page 19 – Long sentence** (≈39 words)

Excerpt: “Family - (Fig S2C) and genus -level (Fig 2D) annotations were dominated by 233
aggregated labels (family “Other” 83.1%; genus “g_Other” 84.9%), whereas 234
recognized methanogenic families such as Methanosarcinaceae (6.4%) an d 235
Metha...”

Suggested edit/action: Family - (Fig S2C) and genus -level (Fig 2D) annotations were dominated by 233 aggregated labels (family “Other” 83.1%; genus “g_Other” 84.9%), whereas 234 recognized methanogenic families such as Methanosarcinaceae (6.4%) an d 235 Methanocorpusculaceae (5.9%) represented minor fractions.

**[57] Page 19 – Long sentence** (≈37 words)

Excerpt: “The species -level 236
assignments (Fig S2D) in Am were largely unresolved (e.g., “Ambiguous_taxa,” 237
8.0%), indicating that most archaeal diversity in Am remains unclassified at finer 238
taxonomic ranks un der the current reference m...”

Suggested edit/action: The species -level 236 assignments (Fig S2D) in Am were largely unresolved (e.g., “Ambiguous_taxa,” 237 8.0%), indicating that most archaeal diversity in Am remains unclassified at finer 238 taxonomic ranks un der the current reference mapping. 239

**[58] Page 20 – Long sentence** (≈65 words)

Excerpt: “At the phylum level (Fig 2C), “Other” accounted for 98.6%, 241
with only trace assignments to Proteobacteria (1.5%) and Euryarch aeota (1.2%). 242
Class - (Fig S2A), order - (Fig S2B), family - (Fig S2C), and genus -level (Fig 2D) 243
...”

Suggested edit/action: At the phylum level (Fig 2C), “Other” accounted for 98.6%, 241 with only trace assignments to Proteobacteria (1.5%) and Euryarch aeota (1.2%). 242 Class - (Fig S2A), order - (Fig S2B), family - (Fig S2C), and genus -level (Fig 2D) 243 annotations were dominated by “Other” or “g_Other” labels (≈98% at each rank), and 244 species -level reads (Fig S2D) were similarly unresolved (“Other,” 98.6%).

**[59] Page 20 – Long sentence** (≈42 words)

Excerpt: “Under the 245
current annotation pipeline and reference set, taxonomic inference for mAc at the 246
order, family, genus, and species levels is therefore not robust. 247
In contrast, wAc exhibited a clear archaeal signature that was co...”

Suggested edit/action: Under the 245 current annotation pipeline and reference set, taxonomic inference for mAc at the 246 order, family, genus, and species levels is therefore not robust. 247 In contrast, wAc exhibited a clear archaeal signature that was consistent across 248 taxonomic ranks.

**[60] Page 20 – Long sentence** (≈41 words)

Excerpt: “Methanomicrobia comprised 252
45.3% of class -level reads (Fig S2A) while family -level profiles (Fig S2C) were 253
dominated by Methanocorpusculaceae (23.6%) and Methanosarcinaceae (21.3%) and 254
their corresponding genera ( Methanocor...”

Suggested edit/action: Methanomicrobia comprised 252 45.3% of class -level reads (Fig S2A) while family -level profiles (Fig S2C) were 253 dominated by Methanocorpusculaceae (23.6%) and Methanosarcinaceae (21.3%) and 254 their corresponding genera ( Methanocorpusculum and Methanosarcina ) as shown in 255 Fig 2D.
