# Supplementary material for: Bacterial-archaeal co-occurrence in honey bee gut microbiomes across host species and management regimes
Source: PLoS One. 2026 Jan 21;21(1):e0339926. doi: 10.1371/journal.pone.0339926 (PMC12822932; doi:10.1371/journal.pone.0339926)
Supplement: Attachment — Submitted filename: Point by point response.doc [file pone.0339926.s006.doc]

For Editor:

This manuscript explores an important and timely topic with potential to advance understanding of microbial mechanisms contributing to colony resilience. However, several methodological and interpretative shortcomings substantially weaken its robustness. The Materials and Methods section lacks sufficient detail regarding experimental design, including the number of colonies per group, biological replication, and whether samples were pooled by colony. The rationale for using an OTU-based approach instead of an ASV workflow is unclear, and appropriate statistical analyses for diversity and ordination (e.g., PERMANOVA) are missing. Functional predictions based on KEGG and COG are interpreted too assertively and should be reframed as hypotheses. The manuscript would also benefit from clearer reporting of percentages, inclusion of negative controls, improved figure readability, and public deposition of raw sequencing data. Overall, while the study addresses a valuable question, substantial revision is required to ensure methodological transparency and strengthen the validity of the conclusions.

***Response:*** *We thank the editors and reviewers for their careful review and constructive suggestions. We have addressed each point raised by the reviewers and revised the manuscript to enhance methodological transparency, adjust explanatory language where appropriate, and clarify limitations arising from the experimental design. The following is a brief summary of substantive modifications and clarifications made to the editorial abstract:*

*1)* *Data availability: all raw sequencing data and processed tables are deposited at CNCB (Project PRJCA048519).*

*2) Experimental design, replication and pooling: We revised the Materials and Methods to explicitly state that DNA was extracted from multiple individual worker bees and subsequently combined into a single pooled sample per group. Specifically, for each group (Am, mAc, wAc) DNA extracts from 10 worker bees per sampled hive were pooled to create one composite library per group (i.e., one pooled sample per group, n = 1 pooled sample). We now clearly acknowledge that this pooled-sample design prevents estimation of within-group variance and therefore precludes tests that require biological replication.*

*3) Rationale for OTU vs. ASV: The Methods now include an explicit rationale for using a 97% OTU-clustering pipeline for these pooled samples. In brief, ASV denoising (e.g., DADA2/Deblur) provides high-resolution error modelling that is most informative when applied across multiple independent biological replicates; with only one pooled sample per group, ASV-derived gains in resolving within-group variation are minimal and would not enable replicate-based inference. We therefore retained a conservative OTU-based workflow for this descriptive study while noting its limitations and our intention to adopt ASV workflows in future studies with true biological replicates.*

*4) Bioinformatics Details and Quality Control: We have supplemented the Methods section with software and database version information and clarified that quality control parameters are now explicitly labeled. Relevant details have been incorporated into the methodology and supplementary materials.*

*5) β-diversity and statistical tests: We explicitly state that, due to sample limitations, distance-based visualizations under the current design are purely descriptive.*

*6)* *Functional predictions and interpretation: Functional inference methods (PICRUSt v1 with KEGG/COG annotations) are now fully reported and functional results are reframed as hypothesis-generating pending shotgun metagenomic/metatranscriptomic validation*

*7) Negative controls and contamination: We acknowledge that extraction/PCR negative controls were not sequenced in this run, describe contamination-minimizing procedures used.*

*8) Figures, tables and reporting of percentages/uncertainty: Figure legends and table captions have been updated to define percentages (relative to total high-quality reads per pooled sample), indicate sample origin (pooled, n = 1), and clarify where uncertainty cannot be calculated.*

*Detailed, point-by-point responses to each reviewer comment and the exact manuscript changes are provided in the enclosed Response to Reviewers document and the revised manuscript.*

*We believe these revisions substantially improve transparency and appropriately temper inferential claims. The manuscript now presents the study as a descriptive, hypothesis-generating comparison of pooled overwintering gut communities and sets out concrete plans for follow-up work. We are grateful for the Editor’s and reviewers’ helpful comments, which have materially strengthened the manuscript.*

For Reviewer #1:

1. Data and Code Availability (mandatory).

Please deposit the raw FASTQ files, sample‐level metadata (group, colony ID, location/GPS, collection date), processed ASV/OTU tables, taxonomic assignments, the phylogenetic tree, and all analysis/figure-generation code (e.g., QIIME2/phyloseq scripts) in a public repository (e.g., SRA/ENA for sequence reads plus Zenodo/OSF for processed data and code) with permanent identifiers (DOIs/accessions). Update the Data Availability Statement to include these links and versions.

***Response:***

*We thank the reviewer for raising this point. All newly generated raw data have been deposited with the China National Center for Bioinformation (CNCB). The accession numbers are listed in the "Data Availability" section of the revised manuscript: Biological project number: PRJCA048519, sample accession numbers SAMC5999114–SAMC5999116; archaea community data: SAMC5999117 and SAMC5999119. The corresponding links and accession numbers have been added to the revised manuscript for easy access by reviewers and readers. Please see line 486-490.*

2. Field permissions and ethics.

For wild *A. cerana* sampling, specify the permitting authority and the permit/application number, and report the sampling locality and dates in both the Methods and Ethics Statement. If a formal permit was not required, please cite the relevant regulation or exemption and state this explicitly.

***Response:***

*We appreciate the reviewers' professional questions.* *At the time of conducting this research, there were no specific regulations governing the care and use of insects in the study. All bees were collected on January 2, 2021, in Daying County, Sichuan Province. Western honeybees (Apis mellifera) and domesticated Oriental honeybees (A. cerana) were obtained from private apiaries in Daying County, while wild A. cerana individuals were collected from natural sites within the same county. The information above has been revised in the updated manuscript. Please see line 113-128.*

3. Sampling design and statistical independence.

Clarify the experimental unit. Please report (i) the number of colonies sampled per group, (ii) the number of worker bees per colony, and (iii) whether samples were processed as pooled or individual libraries. I recommend treating colony as the unit of independence and reporting group-wise n (colonies). If available, include a brief power/precision justification or at least report effect sizes and confidence intervals alongside p-values.

***Response:*** *We have clarified the vague description of the samples.* *For the sequencing samples in this study, we collected 10 worker bees from each group. For Western honeybees, 5 colonies were randomly selected from 200 colonies (excluding diseased and weak colonies), and 2 adult worker bees were randomly chosen from each hive. For managed Eastern honeybees, 5 colonies were selected from 30 colonies, with 2 adult worker bees randomly chosen from each colony. Due to the limited number of wild Eastern honeybee colonies and the difficulty in collecting samples, we only located 3 colonies in areas distant from Western honeybee and managed Eastern honeybee populations. A total of 10 adult worker bees were selected from wild hives. Subsequently, we extracted and pooled the guts of 10 worker bees from different groups to form a single sample. This approach minimized intraspecific variation, allowing for better comparison of interspecific differences. Unfortunately, this method precluded the calculation of confidence intervals and P-values. Nevertheless, it provided insights into gut microbiota differences among species and management practices during wintering. Therefore, we further tempered our inferences in this manuscript. Furthermore, we plan to deepen the findings from this study in future work using approaches such as metagenomics. Please see line 113-146.*

4. Bioinformatics pipeline—modernization and transparency.

Consider replacing 97% OTU clustering with an ASV workflow (e.g., DADA2/Deblur) and report software versions and dates (e.g., SILVA 138.1). In addition to chimera removal (e.g., UCHIME), please detail quality-filter thresholds (maximum expected error, read length, homopolymers), and your normalization strategy (rarefaction vs. compositional methods).

***Response:*** *We agree that the Amplicon Sequence Variant (ASV) method (e.g., DADA2/Deblur) represents the current state-of-the-art and offers higher resolution than 97% OTU clustering methods. We also acknowledge the limitations of OTU methods (e.g., potential merging of distinct taxonomic units and masking of subtle variations). However, given the preliminary exploratory and descriptive nature of this study, our primary objective is to capture macro-level compositional differences among different bee colonies (Am, wAc, mAc) at the phylum and genus levels. The 97% OTU clustering method remains a mature and widely applied strategy for achieving this objective. Furthermore, in this study, each of our three groups (wild A. cerata (wAc), artificially reared A. cerata (mAc), and Apis mellifera (Am)) was represented by only one pooled sample (n=1 per group). Because DADA2 relies on modeling sequencing errors across multiple reads and ideally requires multiple biological replicates to accurately infer variation, it is not well-suited to our design with only one pooled sample per group. In practice, running DADA2 or Deblur on a single sample does not alter the results of qualitative community composition inference. Considering these factors, and given that our primary conclusions (intergroup differences) are robust to the choice of clustering or denoising method, we retained the OTU workflow while acknowledging its limitations.*

*To ensure transparency, we explicitly list all software, versions, databases, and quality control parameters in the “Methods” section. In summary, the processing of raw reads proceeded as follows: 1) Software and versions: We used QIIME 2 (v2021.8) for data processing. OTU clustering was performed using VSEARCH (v2.17.1). Chimerism detection was conducted with UCHIME (via VSEARCH). Classification was based on the SILVA 138.1 database. 2) Quality control: First, we performed quality filtering on the paired-end sequencing data (maximum expected errors per sequence = 1.0, sequences with Q<20 were truncated) and removed sequences containing ≥10 bp homopolymers. We merged paired-end reads with a minimum overlap length of 15 bp and a maximum mismatch rate of 0.1% in overlapping regions. These parameters (minimum overlap length = 15 bp, maximum mismatch rate 10%) are now explicitly documented. Chimeric sequences were detected and removed from the SILVA reference database using UCHIME (sensitive mode). 3) OTU Clustering: Following quality control, VSEARCH was used to cluster the SILVA 138.1 database at 97% sequence similarity. Single OTUs were discarded. These details have been added to the revised Methods section. 4) Normalization: To eliminate effects of library size variation, data were normalized for relative abundance (sum-scaling within each sample). Since each group comprised a single pooled sample, no rarefaction was performed; instead, composition (percentage) normalization was applied. For full transparency, these methodological details (software names and versions, database versions, quality control filters, overlap and mismatch settings, etc.) have been added to the manuscript. We emphasize that applying the ASV workflow to pooled samples preserves the overall pattern (dominant taxa and intergroup differences), meaning reanalysis using ASV would not alter the main conclusions of this study. Finally, we explicitly stated in the Methods section that due to n=1, we did not perform statistical standardization (e.g., rarefaction or compositional correction) as these methods are designed for comparing replicate samples. Our data (as shown in figures) were converted to relative abundance to enable descriptive visual comparisons across the three independent pooled samples. Please see line 165-195.*

5. Beta-diversity and hypothesis testing.

For community dissimilarity, Bray–Curtis and/or UniFrac (weighted/unweighted) are more appropriate than Euclidean distance. Please test group effects using PERMANOVA (adonis) and, where necessary, pairwise contrasts with FDR correction. If ANOSIM is retained, present it as complementary and discuss metric choice.

***Response:*** *We sincerely appreciate the reviewers' expert suggestions regarding statistical analysis. We fully agree that Bray-Curtis/UniFrac is the standard distance metric for microbial community analysis, and PERMANOVA is the preferred method for testing between-group differences. However, we must clarify one of the most critical experimental limitations of this study: our design included only one pooled sample (n=1) per experimental group (Am, wAc, mAc).*

*Without biological replicates within groups, we were unable to calculate within-group variance. Consequently, any statistical significance test relying on replicate samples—including PERMANOVA and ANOSIM—was mathematically infeasible. Our mention of ANOSIM in the original manuscript was a serious oversight, as it is entirely inapplicable when n=1. We have implemented the following key revisions: 1) Removal of statistical tests: All references to ANOSIM statistical tests have been completely removed from the Methods and Results sections. 2) Clear limitation statements: We have explicitly added statements acknowledging the n=1 limitation in both the “Materials and Methods” (Section 2.1) and “Discussion” sections, emphasizing that all results in this study are descriptive. 3) Re-characterizing PCA: In response to the reviewers’ comments, we re-evaluated the presentation of the PCA shown in Figure S3. We emphasize that this plot serves only as an exploratory visualization of overall compositional differences among the three pooled samples and is not intended for statistical inference; this is now explicitly stated in both the figure legend and the main text.With respect to distance metrics, we acknowledge that the Bray–Curtis dissimilarity is widely used in microbial ecology. PCA, however, is intrinsically linked to Euclidean geometry, and therefore visualizes variation in Euclidean space. Given that the principal limitation of our study is the absence of within-group biological replication (each group is represented by a single pooled sample), the constraint on inference stems from the experimental design rather than from the choice of distance metric. For clarity and simplicity we therefore retained a Euclidean distance–based PCA to depict overall structure; we believe this provides an interpretable, descriptive summary under the current study constraints. Please see line 317-319.*

*In future work, we plan to incorporate true biological replicates (using separate pooled samples or multiple independent gut samples per group). Once replicate groups are obtained, we will employ Bray-Curtis distance and weighted UniFrac distance, applying PERMANOVA (with appropriate multiple testing correction for pairwise comparisons) to rigorously test for intergroup differences.*

6. Archaeal annotation and interpretive tone.

Given the high proportion of “Other/Ambiguous” assignments in A. mellifera and managed A. cerana, temper causal claims and emphasize primer/database limitations. Reframe energy-metabolism inferences as hypotheses and, where possible, propose validation (targeted qPCR with archaeal primers, shotgun metagenomics, isotopic/production assays).

***Response:*** *We thank the reviewers for their constructive suggestions. Because of reference-database limitations at the time of analysis, archaeal classification was substantially constrained, leaving many taxa unassigned in both Apis mellifera and managed A. cerana samples. This uncertainty reduces the reliability of downstream functional annotations and may reflect an enrichment of as-yet-uncharacterized archaeal components associated with management practices. In response to the reviewers’ comments, we have moderated our conclusions and now present these results as preliminary and hypothesis-generating. Further work—such as improved reference databases, targeted archaeal surveys, shotgun metagenomics, or culture-based characterization—will be necessary to confirm and extend these observations. Please see line 361-370.*

7. Function inference from 16S.

State the exact tool (e.g., PICRUSt2/Tax4Fun), reference databases and versions, and quality metrics (e.g., NSTI) used for functional prediction. Clearly acknowledge limitations of 16S-based inference and, if feasible, outline plans for orthogonal validation (metagenomic/metatranscriptomic profiling).

***Response:*** *We greatly appreciate the reviewer's valuable comments regarding the transparency of functional prediction. We sincerely apologize for the lack of detailed explanation in the Methods section. We have supplemented the detailed information on functional prediction in Section 2.4 of the Methods section. We have clarified that the specific tool used for inferring functions is PICRUSt, which predicts functions based on 16S rRNA taxonomic annotation. We have also noted the databases referenced: the Kyoto Encyclopedia of Genes and Genomes (KEGG) and the COG (COG) database. We acknowledge that we did not calculate the Newly Sequenced Taxon Index (NSTI) during our analysis workflow. We agree this is a valuable quality metric and have included it as a limitation of this study. We are also aware of the inherent limitations of any functional inference based on 16S rRNA. We fully concur with the reviewer's perspective that 16S-based functional inference carries significant limitations and can only provide a hypothetical perspective on metabolic potential. We wish to clarify to the reviewer that we explicitly and repeatedly emphasized this limitation in the manuscript. For example, we stated at the conclusion of the Results section: As these results were based on marker-based functional predictions, they should be considered as hypotheses of metabolic potential, and validation with metagenomic or metatranscriptomic approaches is recommended." Furthermore, we fully endorse the reviewer's suggestions regarding validation and have explicitly called for such validation in our abstract, discussion, and conclusion sections. For instance, our abstract clearly states: “Future studies should conduct targeted archaeal analyses and multi-omics approaches for validation.” This is further reiterated in the Discussion section. In summary, we will revise the main text to explicitly state the specific tools and databases used, clarify the predictive nature and limitations of functional inferences based on 16S rRNA, and outline our plans for future metagenomic/macrotranscriptomic validation.*

8. Presentation and reporting clarity.

In figure captions, indicate the data source, axis units/scales, and the error metric (SE/SD/95% CI), and specify sample size (n) per group. For relative abundances, define the denominator (e.g., % of total reads). Ensure accessible color palettes and provide full taxon names at first mention in legends. In Table 1, report uncertainty (SE or CI) alongside point estimates.

***Response:*** *We greatly appreciate the reviewers' detailed feedback on the clarity of the figures and tables. We agree with all suggestions and have comprehensively revised all figures (Fig 1-4) and tables (Table 1): Regarding error metrics (SE/SD/CI) and sample size (n): This again relates to the critical limitation of n=1. With only one pooled sample per group, we cannot calculate any measures of variability or uncertainty (e.g., SE, SD, or CI). We have explicitly stated the sample size in all figure* *legends and table titles as: “n=1 pooled sample per group.” For Table 1, we have clearly indicated in the table notes that all α-diversity values represent point estimates from a single sample and thus do not include uncertainty or error margins. For all relative abundance plots (e.g., Fig. 2), we have added the denominator definition to the legend: “Relative abundance (%) based on total effective sequence reads.” We have reviewed and ensured all necessary information is provided in the legends, and that the full taxonomic name is used upon first mention of each unit. We extend our gratitude once again for your valuable time and insightful comments, which have significantly enhanced the scientific rigor of the manuscript. We believe the revised version more clearly communicates our (albeit preliminary) findings and their limitations. Please see Figure legends.*

For Reviewer #2:

This manuscript addresses an interesting and relevant topic that could contribute to a better understanding of the microbial mechanisms underlying colony resilience. However, the study presents several methodological and interpretative weaknesses that limit its overall robustness, particularly in the Materials and Methods section. In particular, the experimental design does not clearly specify the number of colonies and the level of biological replication; the diversity and ordination analyses lack appropriate statistical testing; and the functional inferences based on predictive approaches (KEGG, COG) are interpreted too assertively. From a linguistic standpoint, the text is generally clear, although the readability of the figures should be improved where possible. It would also be useful to provide the raw sequencing data as supplementary material. Overall, this work is promising and potentially valuable, but it requires a major revision to strengthen its methodological soundness and ensure a more cautious interpretation of the conclusions.

***Response:*** *We sincerely appreciated the reviewer's review and positive comments. We have carefully reviewed your suggestions and comments which are very helpful and constructive. The points raised by the reviewer were clarified, as suggested. Please see point by point responses and the revised manuscript for details.*

1. Line 110–115. It would be helpful to indicate here the number of colonies used for each group, or the total number of colonies included in the study.

***Response:*** *We apologize for the vague description of the samples. For the sequencing samples in this study, we collected 10 worker bees from each group. For Western honeybees, 5 colonies were randomly selected from 200 colonies (excluding diseased and weak colonies), and 2 adult worker bees were randomly chosen from each hive. For managed Eastern honeybees, 5 colonies were selected from 30 colonies, with 2 adult worker bees randomly chosen from each colony. Due to the limited number of wild Eastern honeybee colonies and the difficulty in collecting samples, we only located 3 colonies in areas distant from Western honeybee and managed Eastern honeybee populations. A total of 10 adult worker bees were selected from wild hives. Subsequently, we extracted and pooled the guts of 10 worker bees from different groups to form a single sample. This approach minimized intraspecific variation, allowing for better comparison of interspecific differences. Unfortunately, this method precluded the calculation of confidence intervals and P-values. Nevertheless, it provided insights into gut microbiota differences among species and management practices during wintering. Therefore, we further tempered our inferences in this manuscript. Furthermore, we plan to deepen the findings from this study in future work using approaches such as metagenomics. Please see line 113-146.*

2. Line 114. The expression “Italian Carniolan” is too vague and should be clarified more precisely.

***Response:*** *We apologize for our inaccurate description. The Western honeybee species is* *Carniolan, a non-native strain originating from the Alps and Balkan Peninsula regions of Europe, purchased from the Jilin Province Institute of Apicultural Science (Jilin, China). We have revised the corresponding description in the manuscript. Please see line 116-118.*

3. Line 119–123. It should be specified whether pooling was performed separately for each colony.

***Response:*** *We sincerely thank the reviewers for their valuable comments and apologize for any ambiguity in our previous wording. In this study, gut contents from ten bees were pooled to generate one composite sample for each group (Am, n = 1; mAc, n = 1; wAc, n = 1). This pooling strategy was adopted to minimize inter-individual variation and facilitate comparisons across groups. However, we acknowledge that this approach precludes the assessment of within-group variability, making it impossible to calculate confidence intervals or p-values for individual-level effects. Despite this limitation, the pooled-sample design still provides meaningful comparative insights into the gut microbiota differences among species and management conditions during overwintering. All corresponding revisions have been made in the manuscript. Please see line 131-146.*

4. Line 126. Was a blank extraction control used to check for possible contamination?

***Response:*** *We sincerely appreciate the reviewers’ professional suggestions and apologize for any misunderstanding caused by the insufficient clarity of our Materials and Methods section in the previous version. In this study, Am, wAc, and mAc were designed to serve as mutual control groups; therefore, no additional groups were introduced to avoid unnecessary complexity. Moreover, we took rigorous precautions to prevent contamination during both bee collection and gut content extraction. These measures have now been clearly described and updated in the revised version of the Materials and Methods section. Please see line 131-146.*

5. Line 149. The rationale for using an OTU-based approach should be explained.

***Response:*** *We thank the reviewer for this point and agree that the rationale for the OTU-based pipeline should be stated explicitly. Rationale for OTU-based analysis. We acknowledge that Amplicon Sequence Variant (ASV) pipelines (e.g., DADA2, Deblur) currently represent best practice for high-resolution amplicon analysis. In this study, however, each experimental group was represented by a single composite sample produced by pooling DNA from multiple individual bees; thus, the dataset contains one pooled sample per group (n = 1 per group). ASV denoising benefits most from multiple independent biological replicates because error-model estimation and the assessment of low-frequency variants are strengthened by replicate structure. For these pooled, single-sample groupings, a 97% OTU clustering approach (implemented with VSEARCH/USEARCH against SILVA 138.1) provides robust, conservative taxonomic summaries. We emphasize the limitation of the OTU approach (potential merging of closely related taxa) in the revised manuscript and commit to applying ASV-based workflows in future work when additional biological replicates are available. Please see line 117-180.*

6. Line 187. The reported percentage should be explained in greater detail.

***Response:*** *Thank you for the request for clarification. The percentages reported in the Results represent relative abundances (%), calculated from the total number of high-quality sequence reads retained after quality control for each pooled sample. For example, “60.3% of the sequence reads in mAc” indicates that 60.3% of the valid reads in the mAc pooled sample were assigned to the taxon in question. We have clarified in the Methods and in each figure legend that relative-abundance values are expressed as the percentage of total high-quality reads within each pooled sample. Because each experimental group in this study is a single pooled sample (n = 1), these percentages should be interpreted as descriptive summaries of the pooled communities rather than as estimates of inter-colony or within-group variability. Figures and legends have been updated accordingly to make this explicit. Please see line Figure legends.*
